# Australia's regional innovation systems: inter-industry interaction in innovative activities in three Australian territories

**DOI:** 10.1080/09535314.2017.1301886

**Published:** 2017-04-03

**Authors:** Marlies H. Schütz

**Affiliations:** ^a^ Graz Schumpeter Centre, University of Graz, Graz, Austria

**Keywords:** Regional innovation systems, innovation flows, network analysis, input–output analysis

## Abstract

Regional specifics reveal in differences in economic activity and structure, the institutional, socio-economic and cultural environment and not least in the capability of regions to create new knowledge and to generate innovations. Focusing on the regional level, this paper for three Australian territories (New South Wales, Victoria and Queensland) explores patterns of innovative activities in their private business sectors. Furthermore, these patterns are compared to specifics of each region's economic structure. We make use of input–output-based innovation flow networks, which are directed and weighted instead of binary. The value added of the proposed analysis is that we are able to trace a variety of different aspects related to the structure of innovative activities for each territory. It gets evident that mostly innovative activities in each territory are not strong in ‘niche’ branches but in fields of intense economic activity, signalising the high path-dependency of innovative activities in a specific geographical environment.

## 1. Introduction

Even in the globalised world of today, differences in the nature of economic systems do not only prevail *between* countries but also *within* national borders, for instance at the level of regions. These regional specifics reveal in differences in economic activity and structure, the institutional, socio-economic and cultural environment and not least, in the development potential of bounded geographical areas. Beyond, they manifest in the capability of regions to develop new knowledge and to generate innovations. In this context, Porter and Stern (2002) introduced the term ‘innovative capacity’, which – if connected to a regional level – can be defined as a region's

potential […] to produce a stream of commercially relevant innovations. This capacity is not simply the realized level of innovation but also reflects the fundamental conditions, investments, and policy choices that create the environment for innovation in a particular location. (Porter and Stern, 2002, p. 5)

The questions of *how* and *why* regions differ with respect to their innovative capacity have attracted various research in the fields of new economic geography, regional and urban economics, and in evolutionary economics. For instance, concepts such as *learning regions* (Florida, 1995; Morgan, 1997), *innovative milieus* (Maillat, 1995) or the Neo-Marshallian concept of *industrial districts* (Becattini, 2004) made the regional dimension of innovation explicit (see Doloreux and Parto, 2004 for a detailed overview). Within this strand of research in the 1990s the concept of a *regional system of innovation* (RIS) was developed (see e.g. Cooke, 1992; Asheim and Isaksen, 1997; De la Mothe and Paquet, 1998; Howells, 1999 for early contributions). Building on the presumption that regions are an important locus of innovation and that innovation is localised as well as that it is a path-dependent and unique socially and culturally embedded process (Doloreux and Parto, 2004), today the RIS concept constitutes one of the most popular branches of the innovation system framework as emphasised by Cooke (2005).

A textbook example of the existence of regional specifics within a developed and small-open economy is Australia. Due to the existing regional differences, policy strategies to boost regional development have been high on the agenda of both the Commonwealth government and state governments for a couple of years. However, studies in which different aspects of the regional innovation systems (RISs) of Australian territories are examined, to the best of the author's knowledge, are rare. Therefore the motivation of the current paper is to set the focus of analysis on a regional level and to explore for three of Australia's territories – New South Wales (NSW), Victoria (VIC) and Queensland (QLD) – one integral stage of the innovation process, namely innovative activities, as an important aspect of their RISs.

With respect to innovative activities in Australia, Gregory (1993) in an early study on its national innovation system finds a high public funding and a weak private funding to prevail but he emphasised that a changing financing structure is on the horizon. Indeed, things have changed since then: taken together across all three territories on which we concentrate in our analysis, business expenditure on R&D (BERD) hold the largest fraction of more than 60% in gross expenditure on R&D in 2008–2009, which signalises that the private business sector is characterised by rather vivid innovative activities (Australian Bureau of Statistics (ABS), 2010). This empirical finding of a high significance of the private business sector – as one part of the knowledge application and exploitation subsystem (KAES) of a RIS (Autio, 1998) – in carrying out innovative activities further motivates our analysis. Building on the presumption that regions are an important locus of innovation, the current paper seeks to answer the following questions: *Which distinctive patterns of innovative activities do appear in the private business sectors of the three territories? Who are the key actors involved in innovative activities? How does interaction in innovative activities among industries of the same region relate to its economic specifics?*

To answer our research questions we concentrate in our analysis on the meso-level, more precisely, on a sample of 39 industries within each territory. From a methodological viewpoint, to figure out regional economic specifics we calculate location quotients at the industry level. Innovative activities in the private business sector are mapped by regional BERD and we also take account for other sources of funding of innovative activities (e.g. the public sector). We focus on the period 2009–2010 and within an input–output (I/O)-based framework apply the concept of product-embodied innovation flows working on (1) a system of vertically integrated industries and (2) an innovation flow network for each territory. The basic graph-theoretical concept to construct our innovation flow networks is a weighted directed graph and its use we consider as a strong point, as it is not only the direction of interaction in innovative activities which matters but also the intensity of interaction is important for the innovation outcome. Moreover, we apply a range of global and local network metrics to figure out region-specific patterns of innovative activities. This is new in that field of research as they were originally developed in different research areas, for example, econo-physics or world trade analysis.

Through combining the economic dimension with the geographical dimension of innovation, this paper seeks to contribute to a better understanding of regional differences in innovative activities in the private business sectors of the three Australian territories. The identification of the nature of regional innovative activities provides a sound guideline for innovation policy-design in Australia's territories.

The paper is structured as follows: Section 2 contains a brief discussion on the literature background. Section 3 introduces the method used and provides some notes on data handling. In Section 4 empirical results are reported. Finally, Section 5 concludes.

## 2. Literature background

In the 1980s the notion of ‘systems of innovation’ appeared in academic research, with a considerable body of literature having been published in that field since then. Apart from a few unifying elements, such as the need of adopting a systemic perspective for understanding innovation as an interactive learning process (see e.g. Edquist, 2012), the framework has been rather pluralistic from the very beginning on. While in the early years innovation systems were mostly defined along their national borders (e.g. Nelson, 1993), soon studies of innovation systems emerged on different functional and geographical levels. As regards the latter, in the 1990s the concept of a ‘regional system of innovation’ was developed, which since then has evolved into a widely used one. The concept of a RIS has its theoretical foundations in various different research fields such as economic geography, regional and urban economics and evolutionary economics; and as Uyarra (2010) emphasises, it shares some interpretive flexibility.

In the relevant literature, the focus set at the regional level in studying innovation systems has been motivated *inter alia* by the observation that innovative activities are unevenly distributed across space and exhibit a very distinct geography (see e.g. Asheim and Gertler, 2005 for a detailed discussion on the geographical dimension of innovation). Central to the RIS concept is hence the perception that innovation itself is a localised and geographically bounded phenomenon. For defining a ‘region’ in the research context of this paper we follow Cooke et al. (1997, p. 480): the three Australian territories can be termed ‘administrative regions’ and they are characterised by ‘some degree of policy making and political capacity’. Still they are integrated in Australia's national economic structure and institutional environment. In identifying the main building blocks of a RIS Autio (1998) distinguishes between two: (1) the KAES, and (2) the knowledge generation and diffusion subsystem (KGDS). While the former refers to small- and medium-sized firms as well as to big companies, the latter includes basically public sector research institutions and educational institutions, etc. Following this distinction, for each of the three Australian territories, the KGDS can be associated with a distinct set of (semi-)public actors including universities, research laboratories and other scientific and experimental facilities (Australian Government, Department of Innovation, Industry, Science and Research, 2011). In the case of this paper the focus is, however, primarily on the KAES. More specifically, in concentrating on the meso-level that part of the KAES which we take account of are industries in each territory, which are thus considered as being the central actors of innovative activities. The KGDS in our analysis is only captured insofar as we discuss the structure of funding of innovative activities also in the context of the public sector.

A key argument put forth in the literature on RIS is that innovation in different forms, such as the development of new skills or competencies, technological, institutional and organisational innovations involve a strict path-dependency (see e.g. Fagerberg et al., 2008). In the context of RISs it is argued that a historically grown localised pool of firm-specific material and immaterial resources and assets gives rise to specific regional innovative patterns and this is an important source of regional competitive advantages (Doloreux and Parto, 2004). Related to this, a high geographical concentration of specialised knowledge and the formation of clusters lead in turn to the dissemination of positive externalities in the form of knowledge spillovers. The idea of external knowledge spillovers dates at least back to Arrow (1962) and Romer (1986), who discussed them in an intra-industry context, as well as to Jacobs (1969), who focused on knowledge spillovers in an inter-industry context. In the literature on RISs, clustering and potential knowledge spillovers take thus a central role (see e.g. Cooke, 2001; Asheim and Coenen, 2005). Yet, in the research context of this paper we follow a different notion of clustering: Apart from the geographical nature of clustering, this can also be associated with a different reference space, namely the more abstract, ‘economic space of supply and demand’, an idea put forth by DeBresson (1996, p. 151) amongst others. In this case, clustering refers to proximity in the economic space and at the meso-level this implies that the more intense the linkages between any pair of industries, the more they cluster and the closer they are to each other, whereas those industries not or weakly connected are not proximate. As highlighted by Montresor and Vittucci Marzetti (2008), clustering related to the geographical space is therefore not a precondition for clustering in the economic space.

To maintain a systemic perspective on innovative activities in each territory, we apply the concept of product-embodied innovation flows. Different from the disembodied nature of innovation, it is assumed that innovation is of an embodied character which implies that new knowledge spreads through an economy over the existing industry-linkage structure and is embodied in the transfer of commodities between industries. The idea that the existing (industry-)linkage structure of an economy is the basis upon which innovations are generated and spread through the system was already advocated by Marengo and Sterlacchini (1990). Beyond, in the literature on innovation systems the notion that innovations are closely tied to the existing production-and-linkage structure was reinforced for instance by Andersen (2010). Industry-linkages act thus as carriers for ‘new or improved artefacts’, and related to this, the direction of product-embodied innovation flows is highly correlated to the existing flows of commodities between and within industries (Leoncini and Montresor, 2003, pp. 49 ff). Building on the embodied nature of innovation, there has been a considerable body of literature which make use of different innovation input indicators, such as R&D expenditure, to study the relevant ‘technology flows’ (Papaconstantinou et al., 1998), ‘innovation flows’ in vertically integrated industries (Leoncini et al., 1996; Leoncini and Montresor, 2000) or ‘knowledge flows’ (Hauknes and Knell, 2009). More recently, the concept of product-embodied innovation flows was applied by Brachert et al. (2016), who study, based on R&D personnel, clustering patterns at the regional level for the case of Germany. Different from some of these works which rely on binary directed graphs as the basic graph-theoretical concept (e.g. Leoncini and Montresor, 2000; Montresor and Vittucci Marzetti, 2009; Brachert et al., 2016), we work on weighted directed graphs to derive an innovation flow network for each territory. To these regional innovation flow networks we apply a range of network metrics borrowed from other fields of research. Together with a measure of the degree of vertical integration of industries these enable us to characterise regional patterns of innovative activities along the following dimensions: (1) the dependence of an industry on external versus internal sources of R&D; (2) upstream and downstream pervasiveness; (3) whether innovative activities among industries are centralised or rather systemic; and (4) clustering patterns in innovative activities. By applying the concept of product-embodied innovation flows, one assumes that innovations are the outcome of taking conscious effort and further, as they constitute a flow-size rather than a stock-size this implies that one has to assume that the innovation process is instantaneous and complete (Marengo and Sterlacchini, 1990). Still, the concept proves beneficial for our research purpose as it allows doing justice to a systemic perspective on innovation, which is a key presumption in the study of innovation systems. The value added of the adopted concepts and analytical techniques is that we are able to trace a variety of different aspects related to the structure of innovative activities in each territory, which equally can be compared across them. Furthermore, as discussed in more detail below, by combining regional I/O-tables with regional BERD data we do explore innovative activities as an activity which is embedded into a distinct economic and geographical environment.

## 3. Method and data

### 3.1. The regionalisation procedure

We map the production structure of each territory by a single-region open I/O-model. In the I/O-literature different methods exist for regionalising I/O-tables.^1^ Depending on the amount of regional data available, one can broadly distinguish between completely survey-based techniques, partial survey- and non-survey-based techniques. In this paper the choice of a ‘hybrid’ technique seems justified for mainly two reasons: it allows first, incorporating the maximum of available regional data, and second, working on the full regional production structure as the reference space for innovative activities. Hence, apart from domestic regional intermediate and final demand flows also interstate and international trade flows are accounted for. Since the national I/O-table for Australia represents nothing but the total of all regions' production structures, deriving the regional I/O-tables from it seems convenient. The regionalisation technique is found in a bi-proportional optimisation method, called GRAS, which was developed by Junius and Oosterhaven (2003). Basically GRAS, which is an extension of the RAS-algorithm (Stone, 1961), is used for updating I/O-tables. The idea of ‘updating’ can be related to either a time-dimension or, as is the case in this paper, to a spatial dimension.

Following Junius and Oosterhaven (2003, p. 89), the basic information which is required to regionalise the national I/O-table includes a matrix 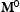 of dimension 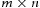, where elements might be positive, zero or negative. Furthermore, it requires an *m*-vector 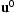 including row-sums of matrix 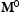 and an *n*-vector 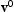 of column-sums of matrix 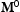 as well as a given *m*-vector of ‘new’ row-sums 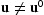 and a given *n*-vector of ‘new’ column-sums 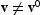. To find from this information set a new updated matrix 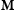, where row- and column-sums are included in the vectors 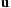 and 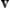, the matrix entries 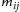 of 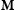 have to be chosen according to:
(1)
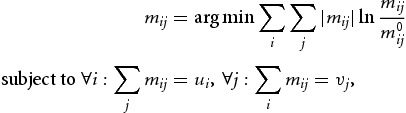
 with 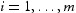 and 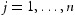. In our case, matrix 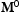 is given by the concatenated matrix version of the national intermediate use and the final use matrix, and 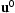 and 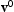 correspond to row- and column-sum vectors of this matrix. The constraining row- and column-sum vectors (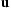 and 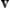) are the regional gross output vectors as well as vectors including total intermediate and final use for each territory at the industry level. Given these basic data, the GRAS algorithm is run and the regionalisation completed. GRAS estimates thus the respective regional matrix through finding a new matrix, which deviates least from the given national matrix and satisfies exogenously given row- and column-sums.

### 3.2. Regional concentration patterns of economic activity

To get an idea about regional economic specifics, we calculate location quotients 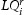 for each industry 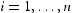 and for each region *r*=1,2,3 (see e.g. Miller and Blair, 2009). These are given by the following formula:
(2)
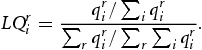
 In Equation 2, 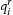 denotes the gross output of industry *i* in region *r*. Values of location quotients strictly greater than 1 signalise that a particular industry has an above-average geographical concentration in a specific region and for a value less than 1 it is only scarcely represented in that region. Hence, location quotients help us to identify specific characteristics of economic activity in each territory.

### 3.3. Regional patterns of innovative activities

#### 3.3.1. A subsystem perspective on innovation flows

As regards the formal description of our single-region I/O-model, for each of the three Australian territories again with *r*=1,2,3 and for *n* industries, let matrix 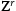 of dimension 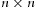 contain intermediate demand (both domestic and imported), expressed in current prices of AUS-$. Furthermore, vector 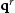 of dimension 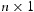 refers again to industry levels of gross output and vector 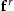 of the same dimension includes total final demand for each commodity in each region. Given these basic definitions, the regional technology matrix 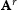 of dimension 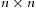 is defined by 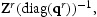 where 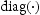 is used as a symbol for the diagonalisation of a vector. A formal description of the well-known open I/O-system for region *r* reads as:
(3)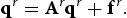 For a given final demand vector, the solution of this system is 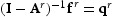, where 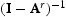 is the regional Leontief-Inverse 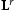 and 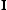 is an identity matrix of dimension 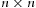.

There are different possibilities to formally link the regional production systems from Equation 3 to innovative activities and to derive the related innovation flow matrices. The option taken in this paper is to construct, in a first step, subsystems (i.e. vertically integrated industries). This allows maintaining the market side as given by final demand, and hence, one obtains a more precise mapping of the structure of supply and demand in each regional economy. The notion of a ‘subsystem’ is based on the idea that the underlying production system can be split into as many subsystems as commodities are produced in total, which in the case treated here corresponds to *n* subsystems. Each of these subsystems produces exactly one commodity as its net output. Of all the other commodities, production within a subsystem equals the amount of means of production used up to satisfy final demand. Formally, the transformation procedure into vertically integrated industries is accomplished by (see e.g. Kalmbach and Kurz, 1985):
(4)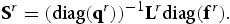 The rows of matrix 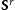, which was first introduced by Momigliano and Siniscalco (1982), include the shares of gross output of industry *i* dedicated to the *j* different subsystems. Therefore, in matrix 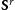 each row sums up to 1. In a next step the regional innovation flow matrix 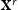 is derived from Equation 4 as follows:
(5)
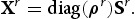


In Equation 5, the vector 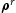 of dimension 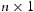 contains regional BERD for the *n* industries and a generic element 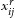 of matrix 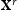 shows the amount of subsystem *i*'s R&D expenditure embodied directly and indirectly in *j*'s final demand.

To make the innovation flow matrix from Equation 5 invariant to scale effects and hence, comparable across regions, in a next step we apply a normalisation procedure. In the related literature different normalisation procedures are usually applied, which however are very sensitive with respect to the empirical outcome.^2^ For the present purpose, a normalisation along columns – through dividing each coefficient of 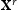 by the respective column-sum – seems justified, as we temporarily focus on structural properties of the single subsystems. Formally, the normalisation procedure of the innovation flow matrix 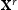 can be written as:
(6)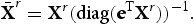 In Equation 6, 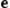 denotes the summation vector of dimension 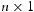 and superscript T refers to the transpose of a matrix or a vector. Comparing then elements on the main diagonal of matrix 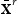 with the sum of elements off the main diagonal, the distribution between intra- and inter-industry innovation flows is made explicit. This provides us with an indicator on whether the R&D content of final demand for a single commodity is determined by innovative activities within the specific industry or whether an industry relies rather on external sources of R&D. Concentrating in a first step on single subsystems in studying patterns of innovative activities proves beneficial as it shows both direct and indirect innovation flows activated by each final demand commodity and hence, this provides a ‘thorough picture of the overall technological interrelatedness’ (Marengo and Sterlacchini, 1990, p. 22) in each territory.

#### 3.3.2. Topological characteristics of regional innovation flow networks

In a next step the subsystem perspective is dropped. Instead we construct, based on the concept of a weighted directed graph (see Appendix B), for each territory an innovation flow network. In our regional innovation flow networks, the set of nodes (also referred to as ‘vertices’) 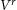 for each region includes its industries, while the set of edges 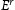 refers to direct inter-industry linkages, again weighted by R&D expenditure. To obtain our networks, this requires for each territory *r* that an adjacency matrix 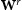 is derived from Equation 5, which is explained in more detail in Appendix B. To explore the internal structure of these networks, we apply a series of network metrics where we distinguish between node-specific indicators and a measure describing the global network structure.

*Upstream and downstream pervasiveness:*^3^ One popular class of node-specific metrics are centrality measures (see e.g. Borgatti, 2005 for an overview). In general, centrality measures provide information about ‘the importance of a vertex in a network’ (Newman, 2004, p. 2). In the following we apply *strength centrality* (see e.g. Fagiolo, 2007 for an application), which constitutes a simple but convenient metric to figure out the importance of single nodes in each regional innovation flow network according to the level of interaction with other industries in innovative activities. Since the adjacency matrix 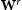 is not symmetric, one has to distinguish between *in-strength* and *out-strength*, defined as follows:
(7a)
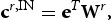

(7b)
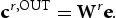

In-strength 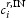 of a single industry *i* refers to direct innovation in-flows, whereas out-strength indicates the level of R&D an industry distributes directly to other industries downstream. The higher the in-strength (out-strength) centrality score for a single node, the more central is its status in the network with respect to the level of R&D in-flows (out-flows). However, while an industry might be central with respect to in-strength, out-strength or both, there is no information whether this stems from a single strong linkage to another industry or from a relatively equal distribution of inter-industry interaction. To check for this property, in-strength (out-strength) is evaluated together with an inequality measure – the column-wise (row-wise) Gini-Index. To calculate it, each column (row) *j* 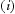 of matrix 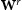 is reduced by the element for which *i*=*j*, which by definition is equal to 0. After that, the *n* column (row) vectors of dimension 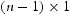 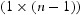 are normalised along column- (row-)sums once again, and elements 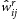 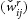 of the normalised column (row) vectors are sorted in ascending order. The respective Gini-Index for a single industry 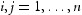 reads as:
(8a)
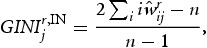

(8b)
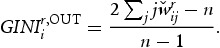


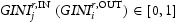
 and the higher its value, the more concentrated are inter-industry innovation in-flows (out-flows) for an industry 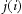. Evaluating strength centrality together with the Gini-Indexes, a high in-strength (out-strength) centrality score together with a 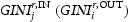 which approaches 0, signalises a high upstream (downstream) pervasiveness of a single node or a high independence from other industries otherwise. Similar to direct linkage analysis (see e.g. Miller and Blair, 2009) this provides us with a measure of backward and forward linkages in innovative activities and we can figure out the key industries in each region which are most connected to other actors with respect to innovative activities.

*Network hierarchy & strength centralisation:* Gini-Indexes calculated in Equations 8a and 8b describe a local property, whereas they do not reveal the hierarchical structure of the whole network. To account for centralisation within the whole network, a distance measure is calculated. Due to the asymmetry of 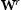, again two versions of this centralisation measure are distinguished. For 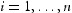 and *r*=1,2,3 the centralisation measure is given by:
(9a)
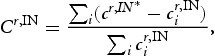

(9b)
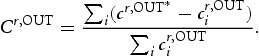


Following partly Freeman (1978–1979),^4^ in the numerator of Equations 9 centralisation is measured with respect to the distance of each node to the most in- (out-)central one denoted by 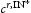 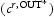. In the denominator we take total in- (out-)strength as the reference point. In the case of *n* nodes, 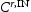 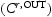 could take values ranging from 0 to 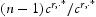. The higher 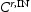 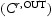, the stricter is the hierarchy within the respective regional innovation flow network regarding the contribution of each single industry to overall innovative activities and the less systemic the network behaves. On the contrary, the lower 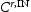 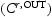, the more heterarchic are innovative activities and the more systemic is the structure of the whole network, signalising a high degree of interaction in innovative activities among industries.

*Clustering patterns in innovative activities:* Opting finally for a measure of clustering patterns, we apply two network metrics, which were initially introduced by Onnela et al. (2005). As discussed before, in the research context of this paper the idea of clustering in innovative activities refers to proximity between smaller groups of industries defined by the economic space as the domain. From a graph-theoretical point of view, we capture ‘smaller groups of industries’ by the concept of a subgraph *g*, for which it holds that its set of nodes 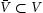 and its set of edges 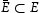 (Harary et al., 1965). In exploring the extent to which ‘tightly connected neighbourhoods’ (Fagiolo, 2007, p. 1) appear within each territory we concentrate on subgraphs *g* of order 3 (i.e. triangles). With respect to the direction of linkages, we are only interested in heterarchic relationships in a triangle, similar to Montresor and Vittucci Marzetti (2008), who test multiple clustering techniques to figure out how innovations cluster in binary directed networks. We concentrate in our analysis thus on triangles which form cycles and in which each node is reachable from any other belonging to the same triangle (i.e. strong components). We depart from Montresor and Vittucci Marzetti (2008) since in the identification of clustering patterns they rely on a subsystem perspective (and account for both direct and indirect innovation flows), whereas our research strategy is to drop the subsystem perspective and to consider only direct innovation flows between industries. For any single regional triangle 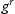, it must hold that 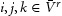 and 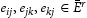, where 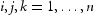 and *r*=1,2,3. To obtain information on the level of interaction within cycles, we first calculate *subgraph-intensity*:
(10a)
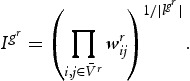
 In Equation 10a, 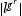 denotes the number of linkages within a particular subgraph 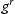, which in the case of studying triangles always corresponds to 3. For any cycle 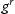, subgraph-intensity counts the weights of edges in the cycle, which is mathematically given by the geometric mean of edges. The higher 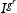, the higher the proximity of actors within a cycle. In addition to subgraph-intensity, it is interesting to know whether interaction within a cycle is also heterarchic with respect to the contribution of each participating node, or whether interaction is rather hierarchical in terms of the weight distribution and therefore only one industry dominates innovative activities within a cycle. To check for this, another network motif called *subgraph-coherence* is calculated:
(10b)
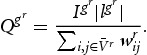


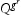
 is defined as the ratio of the geometric to the arithmetic mean of edges within a cycle. For any cycle 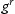, 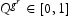, and the higher 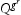, the more equally distributed is interaction within a cluster. On the contrary, for a 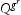 close to 0, interaction within the cycle is relatively unevenly distributed.

### 3.4. Data

Our data set sources from publications of the ABS and basically it includes three pieces: First, regional BERD data for 2009–2010 (ABS, 2013a) and gross R&D expenditure data for 2008–2009 (ABS, 2010); second, the Australian national I/O-table for 2009–2010 (ABS, 2015); and third, state accounts data for 2009–2010 (ABS, 2013b; 2014a).

- Regional BERD are classified according to ANZSIC06 on an industry-subdivision level (2-digit level). ANZSIC06 constitutes the Australian and New Zealand standard industrial classification and the version used in this paper was released in 2006. This classification scheme resembles the NAICS classification but ABS also provides detailed correspondence tables to ISIC. It was decided to work on this in an international comparison somewhat non-standard classification scheme, as definitely national classification schemes represent best national economic specifics and even more so, regional economic specifics. Preparatory steps include the estimation of some single missing values in regional private BERD vectors, through using data of previous and following years. If it is not possible to robustly estimate the missing data from other years, industries are merged and the missing values are calculated as the difference between the industry-division (1-digit level) R&D expenditure and the sum of the corresponding available industry-subdivision level data. Moreover, to obtain information on the KGDS, we also use gross R&D expenditure data at the regional level which shows *inter alia* the public sources of funding of innovative activities in each territory. Note that these data are for the period 2008–2009, which however is the latest available data on this indicator published by ABS.^5^
- Originally, national I/O-tables for Australia are published at the level of IOIG (industry-output-industry-group) which is based on ANZSIC06. Hence, ABS guarantees that both the ANZSIC06 as well as the IOIG are harmonised, such that we could re-classify the national I/O-table according to the available regional BERD data, using as a starting point an aggregated version of 39 industries. Regarding the preparation of national I/O-data for the regionalisation procedure, the national I/O-table where imports are indirectly allocated is taken, since this allows us working on the full technological coefficients and hence, the full production structure is reflected, regardless of the origin of inputs. Further preparatory steps for the regionalisation procedure include first, the merging of the export and the import final demand component to a net-exports vector in the national I/O-table. Second, since the change in inventories final demand component cannot be seriously estimated on a regional level with the available state accounts data, in the national I/O-table this component is added to the net-exports vector. Taking then the intermediate and final use matrix from this slightly modified national I/O-table as well as its row- and column-sum vectors, these serve as the main inputs for the regionalisation procedure.
- Preparing our state accounts data consists of the following steps: first, regional industry-subdivision gross output vectors are derived by weighting the national industry-subdivision gross output vector with regional industry-subdivision employment vectors (ABS, 2013b). Second, ABS (2014a) publishes on an annual basis regional data on gross state product (GSP), total (private and public) final consumption expenditure, total (private and public) gross fixed capital formation as well as total international trade. By definition under the expenditure approach, the GSP is simply the sum of total final consumption expenditure, total gross fixed capital formation, interstate and international net-trade as well as changes in inventories. Given that, the missing totals of regional final use (i.e. interstate net-trade and changes in inventories) are calculated as the difference between GSP and the sum of available regional total final demand components. Third, vectors of regional gross value added on an industry-division level are taken from the state accounts data to derive regional industry-subdivision level gross value added vectors, through weighting them with shares of national industry-subdivision level data in national industry-division level data, following ABS (2014b). Since by definition, intermediate use is the difference between gross output and gross value added, finally regional intermediate use on an industry-subdivision level can be calculated. The obtained regional gross output vectors and regional intermediate and final use vectors serve as another input for the regionalisation procedure.

Furthermore, we make use of a modified version of the Castellacci innovation taxonomy (Castellacci, 2008), which is an extension of the Pavitt innovation taxonomy (Pavitt, 1984). We decided for applying this innovation taxonomy to our industry sample as it integrates both manufacturing and service sector industries within a unified framework doing so justice to the increasing importance of innovations in service sector industries. In the original taxonomy, industries are grouped first to four ‘meta-categories’ and subsequently a refined taxonomy including eight sectors is implemented.^6^ Note that due to specifics of ANZSIC06, only seven sectors of the original Castellacci taxonomy can be distinguished, namely: (1) *knowledge intensive business services* (KIBS), (2) *science-based industries* (SCB), (3) *scale-intensive industries* (SCI), (4) *network infrastructure services* (NIS), (5) *physical infrastructure services* (PIS), (6) *supplier-dominated goods* (SDG) and (7) *supplier-dominated services* (SDS). To account for the fact, first, that mining industries and the agriculture sector are important for Australia's economy and second, some parts of the country are rich in some natural resources (e.g. mineral resources) while there is shortage of others (e.g. water) in some parts of the country, we introduce another sector including *energy and resource-based industries* (ERB). We do so since this might give rise to region-specific innovative activities in these industries. Furthermore, we group some service and goods industries which are not covered by the Castellacci taxonomy to *other industries* (OI). Single industries are classified to the nine sectors based on correspondence tables (ABS, 2008) between ANZSIC06 and ISIC Rev. 3.1 as shown in Appendix A. If only partial correspondence between economic activities under both classifications is given, the main field of economic activity is used as the decisive criteria to allocate a particular industry to the respective Castellacci sector.

## 4. Empirical results

### 4.1. Regional economic structures

We start the discussion of our empirical findings with an illustration of regional economic specifics. With an economy-wide industry gross output of 764.148 billion AUS-$ in 2009–2010, NSW in terms of the size of its economy is the largest region, contributing more than 30% to Australia's economy-wide gross output. This is followed by VIC, generating 597.684 billion AUS-$ of gross output and a share of about 24% in the Australian gross output. Compared to the other two regions, QLD's gross output is lowest in 2009–2010, with 511.275 billion AUS-$ and a share of nearly 21% in economy-wide gross output. Altogether, these three territories are key regions in the Australian economy and their development potential as well as competitiveness proves to be highly decisive for the whole Australian economy.

Values of average sectoral location quotients are presented in Table 1 and in all three territories several differences in production systems appear: Especially SDS, NIS and more knowledge intensive service branches, namely KIBS, are highly localised in NSW. In VIC KIBS perceive of an even higher average location quotient and it is particularly the ICT-industry *Computer System Design and Related Services* which is highly geographically localised in the territory, while in NSW both KIBS industries *Professional, Scientific and Technical Services* and *Computer System Design and Related Services* have a location quotient greater than 1. A leading position of KIBS in VIC and NSW indicates that the territories perceive of a strong supporting knowledge base, which, however, does not apply to QLD. A high geographical concentration of NIS in NSW, which is driven by the industries *Finance* and *Insurance and Superannuation Funds, Auxiliary Finance and Insurance Services* definitely makes sense, since the territory hosts Australia's financial hub – Sydney. Remarkably, just a single sector including only manufacturing industries, namely SCB, perceives of an above-average concentration in NSW, signalising that its economy is strongly oriented towards service sector industries. Interestingly, in VIC multiple sectors are represented with an average location quotient strictly greater than 1, suggesting that the region is competitive in various branches – including both manufacturing and service sector industries; especially SCB, SCI and SDG are highly localised in a regional comparison. Compared to the other two territories, in QLD economic activity is particularly strong in ERB and OI. Within the first sector it is the *Mining* industry which drives the average sectoral location quotient and regarding OI, *Heavy and Civil Engineering Construction* and *Construction Services* perceive of an extraordinarily high geographical concentration. In these industries there prevails thus a comparative advantage within the territory. Noteworthy, in QLD on average none ‘pure’ service sector plays an outstanding role for its regional economy and compared to the other two territories, especially KIBS are relatively under-represented and these industries are niche branches of its regional economy.

**Table 1.** Average sectoral location quotients in the three territories, 2009–2010.

	NSW	VIC	QLD
ERB	0.866	0.98	1.223
KIBS	1.07	1.096	0.783
NIS	1.136	0.991	0.806
PIS	0.983	1.053	0.964
SCI	0.818	1.321	0.897
SCB	1.067	1.255	0.601
SDG	0.97	1.2	0.811
SDS	1.165	1.019	0.731
OI	0.976	0.966	1.075

Note: Based on ABS Data (2013b; 2014a; 2015). Author's own calculations.

A considerable variation in economic activity and differences in the geographical concentration of industries and sectors across regions show that each territory disposes of its unique production structure. Given this finding, we investigate in a next step, how this relates to patterns of innovative activities in each region.

### 4.2. Regional patterns of innovative activities

**Figure 1.**
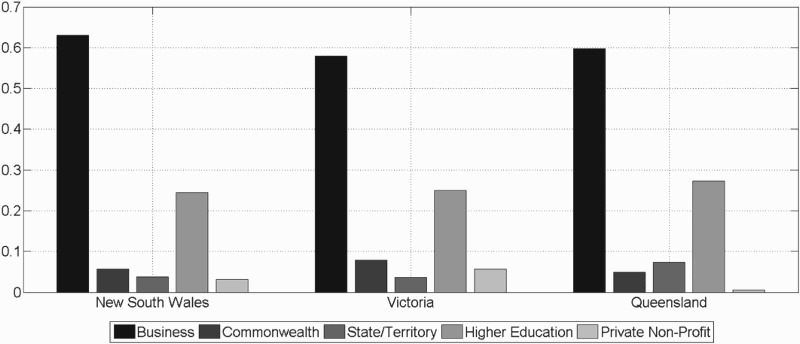
Share of each sectoral source of funding in gross expenditure on R&D in the three territories, 2008–2009.

Note: Based on ABS Data (2010).

*The structure of funding of innovative activities:* As illustrated in Figure 1, with respect to the structure of financing of innovative activities in each territory, the level of gross R&D expenditure in 2008–2009 was highest in NSW with a value of about 8.3 billion AUS-$. In that period in VIC about 7.1 billion AUS-$ and in QLD only about 3.9 billion AUS-$ were spent in total (ABS, 2010). As to the sources of funding of innovative activities, in each of the three territories a similar structure prevails: across all three territories, BERD hold the largest fraction in gross expenditure on R&D, which signalises, that the KAES is characterised by rather vivid innovative activities. This could be the outcome of a specific innovation policy run by the Commonwealth government since 1985, namely a generous Tax Concession Scheme, which was replaced by the R&D Tax Incentive Scheme in 2011. Especially a high continuity in this incentive scheme has fostered innovative activities in the private business sector, which is supported by the fact that the number of businesses which registered for the Tax Concession Scheme has continuously increased since its implementation date (Australian Government Department of Industry and Science, 2010).

Another similarity across regions is found with respect to the higher education sector, which accounts for the second largest source of funding of innovative activities in 2008–2009. Hence, universities, research laboratories and other scientific and experimental facilities, which can be associated with the KGDS, are key actors in each territory's RIS. The funding of innovative activities by public sources is however less significant: across all three territories direct public R&D funding is comparatively low, whereas indirect public R&D funding through the Tax Concession Scheme provides a more effective incentive for businesses to innovative. Different from QLD, in NSW and VIC a relatively larger amount of government funding stems from the Commonwealth government and state governments only play a minor role in funding innovative activities. Given the finding of a relatively homogeneous distribution of sources of funding of innovative activities across regions, we next focus on the private business sector. Digging yet deeper, we seek to identify whether at the level of industries a more heterogeneous picture prevails across regions in terms of interaction in innovative activities.

*Intra- versus inter-industry innovative activities:* As can be seen from Table 2, a common picture across all regions emerges for KIBS and SCB, which are on average strongly vertically integrated compared to other sectors and there is hardly any intra-sectoral variation in results. These empirical findings confirm a stylised fact of the Castellacci taxonomy as KIBS and SCB have themselves strong innovative capabilities and are actively innovating, relying primarily on internal sources for creating new knowledge. Together with previous results of location quotients, the high geographical concentration of SCB in VIC, and of KIBS in NSW and VIC goes thus alongside with a strong internal capability to innovate. Still, even the niche position of KIBS in QLD's economy is accompanied by strong internal innovative activities. Across all regions on the other extreme OI can be found which on average are heavily dependent on external innovative activities and where the R&D content of final demand is mostly determined by other industries upstream, which is also true for downstream service sector industries (i.e. SDS). With respect to their counterpart in manufacturing (i.e. SDG), in QLD and NSW these industries perceive of slightly higher internal capabilities to innovate than in VIC. Regional differences are also observed, first, in SCI which are more strongly vertically integrated in NSW compared to QLD and VIC. Second, the R&D content of NIS final demand goods is less driven by external sources in NSW and VIC compared to QLD. For the second group of supporting infrastructure services (i.e. PIS) regional differences are more modest. Third, ERB in QLD build by far more on internal sources of innovative activities than is the case in NSW and VIC. Regarding these differences across regions, one has to bear in mind that there is some variation in single industry-values within these sectors. For instance, for NSW's NIS it is particularly *Finance* which perceives of a high degree of vertical integration and which drives the sectoral result. And also in QLD's ERB, *Mining* contributes strongly to the high average degree of vertical integration in this sector. Together with results of location quotients in NSW and QLD, this indicates that in both territories a comparative advantage in these industries goes hand in hand with a high potential to create new knowledge internally and the R&D content of final demand is to a good deal driven by internal innovative activities.

**Table 2.** Average sectoral degree of vertical integration in the three territories, 2009–2010.

	NSW (%)	VIC (%)	QLD (%)
ERB	35.8	39.7	52.3
KIBS	71.5	73.5	81.6
NIS	52	53.7	29.8
PIS	37.7	33.8	33.1
SCI	49.9	43.6	41.8
SCB	64.2	74.9	67.8
SDG	42.4	37.3	42.1
SDS	28.1	31.1	31.6
OI	19.3	28.6	24.8

Note: Based on ABS Data (2013a; 2013b; 2014a; 2015). Author's own calculations.

*Network hierarchy & strength centralisation:* Dropping the subsystem perspective, we next look at the global structure of each regional innovation flow network. The centralisation-indexes reported in Table 3 reveal the most hierarchic structure in NSW, showing that inter-industry interaction within its RIS is least systemic. For the other two territories the following is observed: while QLD is second ranked in a comparison across regions in terms of the level of both centralisation-indexes, innovative activities are most heterarchic in VIC. This suggests that in VIC a systemic structure in the innovation flow network prevails and there is a comparatively equal distribution of innovative activities in its private business sector. On the contrary, innovative activities in the other two territories are more bound to a few single actors of their KAES.

**Table 3.** Strength centralisation within the three territories in 2009–2010.

	In-centralisation	Out-centralisation
NSW	10.603	13.884
VIC	4.711	5.238
QLD	5.272	7.056

Note: Based on ABS Data (2013a; 2013b; 2014a; 2015). Author's own calculations.

As can be further seen from Table 3, out-centralisation across all regions is higher than in-centralisation and there is thus a wider centrality gap with respect to out-strength centrality than in-strength centrality. Put differently, especially the horizontal distribution of regional innovation flows between industries exhibits a relatively strict hierarchy, where only few industries are characterised by strong innovation out-flows in a system-wide context. This can be considered as harmful to the degree of interaction in innovative activities. A similar finding was made by Leoncini and Montresor (2003), who investigate Australia's technological system in the early and mid-1980s as well as in the early 1990s.

Given the existence of several differences in the global network structure across the three territories, we zoom into our regional innovation flow networks and explore further characteristics of region-specific innovative activities.

*Upstream and downstream pervasiveness:* Related to in- and out-strength centrality scores and the corresponding Gini-Indexes, both similarities and differences across regions appear, as can be seen from Table 4, and from Tables C1 and C2 (Appendix C).

**Table 4.** Average sectoral strength centrality scores and average Gini-Index (figures in brackets) for the three territories, 2009–2010.

	Average in-strength			Average out-strength		
	NSW	VIC	QLD	NSW	VIC	QLD
ERB	0.0119	0.0063	0.0091	0.0157	0.0095	0.0679
	(0.851)	(0.801)	(0.804)	(0.801)	(0.807)	(0.773)
KIBS	0.0064	0.0076	0.0057	0.078	0.0992	0.1331
	(0.814)	(0.754)	(0.808)	(0.727)	(0.726)	(0.769)
NIS	0.0807	0.041	0.0434	0.0976	0.366	0.0124
	(0.896)	(0.836)	(0.86)	(0.715)	(0.718)	(0.752)
PIS	0.0302	0.0433	0.0375	0.0311	0.0348	0.0266
	(0.721)	(0.714)	(0.789)	(0.585)	(0.599)	(0.629)
SCI	0.0058	0.0074	0.0099	0.0131	0.0233	0.0245
	(0.839)	(0.789)	(0.894)	(0.772)	(0.774)	(0.801)
SCB	0.013	0.0092	0.0103	0.0317	0.0594	0.0279
	(0.777)	(0.707)	(0.883)	(0.646)	(0.656)	(0.68)
SDG	0.005	0.0055	0.0054	0.0101	0.0107	0.0067
	(0.737)	(0.69)	(0.759)	(0.775)	(0.783)	(0.798)
SDS	0.014	0.0201	0.0149	0.0021	0.0045	0.002
	(0.795)	(0.765)	(0.755)	(0.691)	(0.696)	(0.736)
OI	0.0336	0.0389	0.0453	0.005	0.0083	0.0094
	(0.762)	(0.705)	(0.765)	(0.728)	(0.728)	(0.772)

Note: Based on ABS Data (2013a; 2013b; 2014a; 2015). Author's own calculations.

It is noteworthy, that both in VIC and QLD KIBS industries perceive of the highest average out-strength centrality and also in NSW they are ranked second in a sectoral comparison, whereas with regard to the Gini-Index of innovation out-flows they are found in the middle in each region. Looking more closely at the industry level, both KIBS industries *Professional, Scientific and Technical Services* and *Computer System Design and Related Services* rank among the top-5 out-central ones in each territory. In each territory it is *Professional, Scientific and Technical Services* which has a below-average Gini-Index and which is thus highly connected to other industries downstream. Together with results from the distribution between intra- versus inter-industry innovative activities, our findings show that *Professional, Scientific and Technical Services* is not only strongly innovative itself but its pervasive nature together with a high geographical concentration makes it a central actor of the KAES in NSW and VIC. Even if it is sparsely geographically located in QLD, also there it has a strong distributive impact on other industries downstream.

Another exceptionally high average sectoral out-strength centrality score is observed for QLD's ERB, which is accompanied by a medium-high average Gini-Index. However, regarding out-strength centrality scores of single industries within that sector, there is little homogeneity. Again it is *Mining* which is the most out-central one and this is accompanied by a below-average Gini-Index. Our observation reinforces previous results, that this industry is a central actor in QLD's overall innovative activities and has a deep impact on industries located downstream as it is highly pervasive.

Different from the other two territories, SCB in VIC have a rather high average out-strength centrality score and a low average Gini-Index. Moreover, both industries of that sector, namely *Basic Chemical and Chemical Product Manufacturing* and *Machinery and Equipment Manufacturing* equally contribute to the average sectoral result. The pervasive nature as well as a strong vertical integration of these industries suit the Castellacci taxonomy, since SCB industries are expected to share a high potential to create new knowledge internally and at the same time act as important carriers of innovation flows as they are intensely connected to industries downstream.

Regional differences do also appear with respect to NIS, which in NSW on average have the highest out-strength centrality score. In NSW this high average out-strength centrality score again is driven by *Finance* which is located at the top in the region. Yet, it perceives of an above-average Gini-Index and is thus rather independent, sharing only a few strong linkages to other industries downstream. An outstandingly high out-strength centrality together with a below-average Gini-Index is also observed for the PIS industry *Wholesale Trade*, which, however, behaves similar in a regional comparison, and in QLD this also holds for another PIS industry, namely *Transport, Postal and Warehousing*.

With respect to in-strength centrality scores and the corresponding Gini-Indexes, our findings reveal that there is less heterogeneity across regions. In each territory NIS, OI and PIS have the highest average in-strength centrality scores and while NIS and PIS perceive of a relatively high Gini-Index, OI have a low average Gini-Index. This shows that the latter are highly upstream pervasive, whereas NIS and PIS are rather independent. In each territory the leading industry in in-strength centrality is the NIS industry *Rental, Hiring and Real Estate Services* and for this industry an above-average Gini-Index is observed, confirming that it absorbs innovative activities from only a few other industries upstream. In the OI sector, particularly *Health Care and Social Assistance* as well as *Administration Services and Support Services & Public Administration and Safety* qualify as being heavily dependent on other industries upstream in innovative activities. For PIS, the sectoral result of a high in-strength centrality score is driven by *Wholesale Trade* in NSW and QLD, and by *Transport, Postal and Warehousing* in VIC. Moreover, the SDS industries *Retail Trade* and *Accommodation and Food Services* in each territory perceive of high in-strength centrality scores and a below-average Gini-Index, which indicates a high connection to other industries upstream. Having highlighted some of the most striking results of regional differences and similarities with respect to the pervasive power of industries we next identify clustering patterns of innovative activities.

*Clustering patterns in innovative activities:* In each regional innovation flow network with *n* nodes, a single node *i* engages in a maximum number of 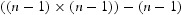 cycles with a non-zero subgraph-intensity. Therefore in our regional innovation flow networks with 39 nodes, in each there exist a maximum number of 18,278 different cycles. In order to deal with this large number, we impose two restrictions in the interpretation of our results. To qualify for the analysis, for a single cycle it must hold that first, subgraph-intensity is strictly greater than the expected subgraph-intensity which would prevail if there was a uniform distribution within a single network (and each edge would have the same share in overall inter-industry innovative activities). Second, subgraph-coherence within a cycle must be strictly greater than the average subgraph-coherence of all existing cycles within a single network.

In a regional comparison it is VIC where the number of triangles which satisfy both restrictions with a value of 548 is highest. This reinforces results observed for the global network hierarchy, where also in VIC the most systemic structure prevails. Furthermore, in NSW 206 cycles and in QLD 219 cycles qualify for our selection criteria.

**Figure 2.**
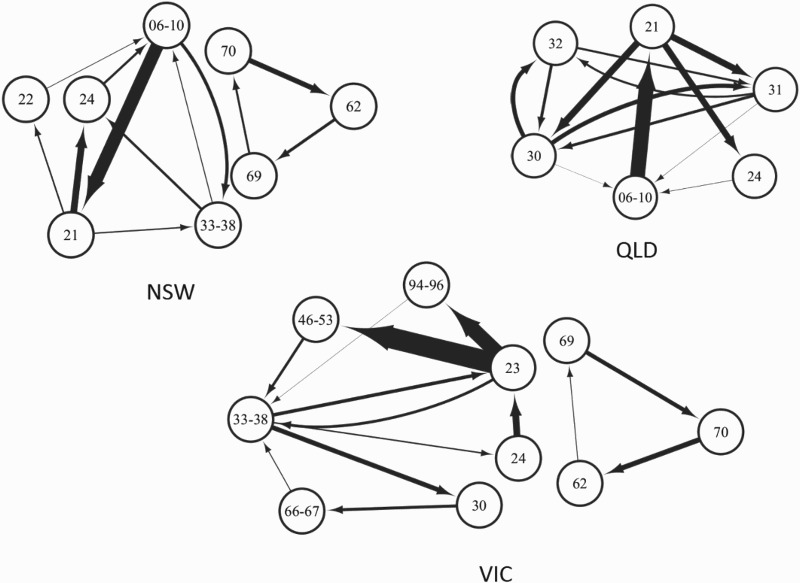
Top-5 clusters in NSW, VIC and QLD, 2009–2010. Note that the width of arrows reflects the weight of the linkage and this is also amenable for a comparison across regions. Clusters have been illustrated by using the open source software Cytoscape. Based on ABS Data (2013a; 2013b; 2014a; 2015).

Note: Author's own calculations.

Looking more closely at the top-5 cycles with respect to the level of subgraph-intensity and -coherence, as illustrated in Figure 2, this gives a good impression about the existing clustering patterns in each territory. As regards the composition of the top-5 clusters, the highest number of nodes participating within them is measured in VIC, followed by NSW, whereas in QLD comparatively few nodes are involved. In QLD the top-5 clusters together form only one strict component and on the contrary in VIC and NSW, two strict components appear.

From a sector-level perspective it is again VIC, where the most systemic structure prevails – industries involved within the top-5 clusters belong to six sectors, and only ERB, SDS and SDG do not participate. Interaction between *Transport Equipment Manufacturing* (23), *Machinery and Equipment Manufacturing* (24), *Transport, Postal and Warehousing* (46–53) and *Wholesale Trade* (33–38) accounts for the two highest subgraph-intensities in VIC. A high participation of industries belonging to different sectors in VIC supports our previous results that the region's diversified and stable industry-linkage structure encourages interaction in innovative activities among complementary industries. Interaction between the two KIBS industries *Professional, Scientific and Technical Services* (69) and *Computer System Design and Related Services* (70) with *Finance* (62) is also comparatively strong in VIC, similar to the situation observed in NSW. Yet, in both regions this cycle is self-contained among the top-5 clusters and no interaction with the rest emerges which might constrain possible knowledge spillovers to other industries.

Surprisingly, the leading cycle and the cycle which is third ranked in NSW with respect to subgraph-intensity are constituted by the ERB industry *Mining* (06–10), the SCI industry *Primary Metal and Metal Product Manufacturing* (21), the SCB industry *Machinery and Equipment Manufacturing* (24) and the PIS industry *Wholesale Trade* (33–38). Apart from the SCB and the SCI industry, NSW in none of the two remaining industries perceives of a location quotient greater than 1. This indicates that clustering patterns in NSW are also driven by industries which form ‘niche branches’ and are not geographically highly localised. Still, by taking the economic space as the domain for defining proximity, specialisation patterns in innovative activities in these industries are evident.

Supporting previous findings is the observation that in QLD innovative activities of service sector industries only play a minor role compared to the other two territories. Apart from *Construction Services* (32), no other service sector industry is involved in its top-5 clusters but the participating industries belong to ERB, SCB, SCI and OI. The highest subgraph-intensity in QLD is measured between a triangle of the ERB industry *Mining* (06–10), the SCI industry *Primary Metal and Metal Product Manufacturing* (21) and the SCB industry *Machinery and Equipment Manufacturing* (24). *Mining* (06–10) participates also in two other of its top-5 clusters and also the OI industries *Building Construction* (30) and *Heavy and Civil Engineering Construction* (31) are strongly represented among the top-5 ones. Thus, similar to NSW, clustering patterns and possible knowledge spillovers in QLD in some cases involve industries which are not geographically concentrated.

## 5. Conclusion

The aim of this paper was to explore distinct patterns of innovative activities within New South Wales' (NSW's), Victoria's (VIC's) and Queensland's (QLD's) private business sector as an integral part of their regional innovation systems (RISs), and in particular of the knowledge application and exploitation subsystem (KAES). Within the proposed analysis we were able to discover for each region the key actors of innovative activities and through concentrating on the industry-linkage structure, innovation was considered as an interactive, rather than an individualistic process. This we consider a crucial issue in studying innovation systems. We further related characteristics of innovative activities in each territory to regional economic specifics adding so another dimension to our analysis of this single stage of the innovation process. Given the presumption that Australia constitutes a textbook example for regional specifics within a developed country, we could show that there are not only specifics of innovative behaviour among different industries within a single region but also that the behaviour of one and the same industry varies across the three territories. This finding substantiates the need of adopting a regional perspective in order to not blur these regional specifics, which might be the case otherwise, if sticking to a higher geographical level of analysis.

The analytical tools which were combined for our research purpose proved helpful in various aspects: By working explicitly on weighted directed graphs based on a single-region I/O-framework and the concept of product-embodied innovation flows, this allowed us studying the topological characteristics of innovative activities among industries without blending the empirical structure. Through applying a variety of metrics we achieved to shed light on different characteristics of innovative activities in the three territories' RISs: First, we were able to uncover the degree to which innovative activities originate from within industries or are driven externally. By dropping in a next step the subsystem perspective we could figure out both network characteristics on a global as well as on a local level. As argued, information on the network hierarchy, on the nature of upstream and downstream pervasiveness of industries reveal the key actors of each territory's private business sector. Regarding the applied clustering technique, this gives a simple but convenient tool to identify industry clusters of specialisation in innovative activities, taking instead of the geographical the economic space as the reference space. For figuring out this latter aspect of interaction in innovative activities, the clustering technique allows to break down an otherwise maybe too complex structure into its most important components. All in all, the analytical techniques which we brought together for our research purpose complement the existing literature on RISs: they enabled us to focus on a variety of different aspects related to the structure of innovative activities in each RIS. Such an analytical framework seems promising as structural characteristics can be properly compared across different geographical locations, which, if one thinks for example of qualitative case studies on RISs, is not always that easy.

Even if we addressed our research question only from a static time perspective, the suggested analytical framework – in case of a more comprehensive and less restricted data set than the one we were imposed to work with – could be extended to an intertemporal framework in future research. Then innovative activities could be explored not only in reference to the existing industry-linkage structure but one could concentrate on its evolution. Another aspect we would like to add is that in our empirical analysis the focus was on the KAES, as one subsystem of the RIS of each territory, or more precisely, on the private business sector. For future research it would be worthwhile to look more closely, than we did, at the role of the knowledge generation and diffusion subsystem in each RIS.

As regards potential policy implications, empirical results have shown that NSW is heavily oriented towards service sector industries and especially knowledge intensive branches are key actors of its RIS. In this respect, we conclude that the significance of these branches in creating new knowledge and in innovating proves favourable for the territory's development potential since productivity and employment growth, in particular of high-skilled labour, in developed countries are highly dependent on the innovative capability of service sector industries. For QLD we found evidence that the focus of innovative activities in the territory is on the Mining industry, which is also a key actor of its regional economy. From a policy-oriented viewpoint, the central position of the Mining industry in QLD signalises that resource-based branches can be still of importance in an otherwise merely service-based economy, as is the case for Australia. Innovative activities in VIC's private business sector are characterised by the most systemic nature compared to the other two territories. The territory is strong in various different branches with respect to economic activities and it innovates in multiple industries. Beyond, the prevailing industry-linkage structure in this territory constitutes a favourable basis for interaction in innovative activities since there are many channels through which innovation flows disseminate through the system. Notably, a high intensity of innovative activities in several manufacturing industries proves beneficial with regard to the development of new key manufacturing technologies, especially associated currently in the US and Europe with ‘smart industry’ and ‘Industry 4.0’. We further observed that in most cases innovative activities in each of the three territories are particularly intense not in ‘niche’ branches but in fields where the respective region already perceives of intense economic activity signalising the high path-dependency of innovative activities in a specific geographical environment. Only for single cases, innovative activities are outstandingly strong in regional ‘niche’ branches. Based on the empirical evidence we conclude that it is by far not the amount of business expenditure on R&D that is spent in a single industry which is decisive for the potential to generate innovations. Rather, we consider it as necessary to adopt a systemic perspective and to concentrate on the interaction structure in innovative activities in order to figure out promising key innovative branches. Following Hirschman (1958), industrial and innovation policy in each region should be targeted at ‘key industries’, implying that one needs to boost further interaction in innovative activities particularly in those industries which share a strong degree of pervasiveness. What holds not only true for the Australian case, but may be translated to other cases, is that the existence of differences in economic structures and in innovative activities, etc. at the level of regions requires a differentiated regional policy approach. Hence, it seems not promising to impose ‘one size fits all’ industrial and innovation policies. Preferably, policy-makers should adopt a differentiated regional policy approach, whereby the regional policy model is developed based on the consideration of the existing regional specifics.

## Supplementary Material

Supplement_Material.pdf

## Footnotes

1 See e.g. Miller and Blair (2009) for a detailed discussion on the different analytical techniques to regionalise I/O-tables.

2 For a detailed discussion on the various normalisation procedures of innovation flow matrices see Montresor and Vittucci Marzetti (2009).

3 See also Strohmaier (2014) for a comprehensive study of upstream and downstream pervasiveness in the context of general purpose technologies.

4 See e.g. Leoncini et al. (1996) or Leoncini and Montresor (2000) for an application of a similar centralisation measure to a binary directed network.

5 We are well aware of the fact that R&D expenditure provides only a proxy for innovative activities. However, as shown in a recent research paper by Hassan et al. (2015) there is a high positive correlation between R&D, as an input indicator for innovation, and patent applications as well as the number of trademark applications filed, as innovation output indicators, for the Australian case. This clearly signalises the importance of R&D for innovation.

6 Based on a two-dimensional taxonomic model, industries are classified to different sectors, where between first, their function in an economic system as determined by their position along the vertical chain, and second, their technological content is distinguished (Castellacci, 2008). To test the validity of the suggested taxonomy, Castellacci (2008) does a comprehensive statistical analysis of CIS and OECD STAN data for a sample of 24 European countries and provides ample empirical evidence in favour of it.

## Appendix

**Table A.1.** Merged Industry Classification based on ANZSIC06.

Industry	ANZSIC06	ISIC Rev.	Castellacci Taxonomy
	Code	3.1 Code	(modified)
Agriculture, Forestry and Fishing	01-05	01-05	ERB
Mining	06-10	10-14	ERB
Food Product Manufacturing	11	15	SDG
Beverage and Tobacco Product			
Manufacturing	12	15, 16	SDG
Textile, Leather, Clothing and			
Footwear Manufacturing	13	17-19	SDG
Wood Product Manufacturing	14	20	SDG
Pulp, Paper and Converted Paper			
Product Manufacturing Printing			
(including the Reproduction of Recorded Media)	15-16	21-22	SDG
Petroleum and Coal Product Manufacturing	17	23	ERB
Basic Chemical and Chemical			
Product Manufacturing	18	24	SCB
Polymer Product and Rubber			
Product Manufacturing	19	25	SCI
Non-Metallic Mineral			
Product Manufacturing	20	26	SCI
Primary Metal and			
Metal Product Manufacturing	21	27	SCI
Fabricated Metal Product Manufacturing	22	28	SCI
Transport Equipment Manufacturing	23	34-35	SCI
Machinery and Equipment Manufacturing	24	29-32	SCB
Furniture and Other Manufacturing	25	36	SDG
Electricity Supply	26	40	OI
Gas Supply; Water Supply, Sewerage			
and Drainage Services; Waste Collection,		40,	
Treatment and Disposal Services	27-29	41, 90	OI
Building Construction	30	45	OI
Heavy and Civil Engineering Construction	31	45	OI
Construction Services	32	70,45	OI
Wholesale Trade	33-38	50-51	PIS
Retail Trade	39-43	50,52	SDS
Accommodation and Food Services	44-45	55	SDS
Transport, Postal and Warehousing	46-53	60-64	PIS
Publishing (except Internet			
and Music Publishing)	54	22,74,72	SDS
Broadcasting (except Internet)	56	92	SDS
Internet Publishing and Broadcasting;			
Internet Service Providers, Web Search Portals			
and Data Processing Services	57, 59	72	SDS
Motion Picture and Sound Recording Activities;			
Telecommunication Services; Library and Other	55, 58, 60	64, 72,	
Information Services		74, 92	NIS
Finance	62	65	NIS
Insurance and Superannuation Funds,			
Auxiliary Finance and Insurance Services	63-64	66-67	NIS
Rental, Hiring and Real Estate Services	66-67	70-71	NIS
Professional, Scientific and Technical Services			
(Except Computer System Design			
and Related Services)	69	73-74	KIBS
Computer System Design and Related Services	70	72	KIBS
Administration Services and Support Services;	72-73,		
Public Administration and Safety	75-77	74-75	OI
Education and Training	80-82	80	OI
Health Care and Social Assistance	84-87	85	OI
Arts and Recreation Services	89-92	92	SDS
Other Services	94-96	93-97	OI

Note that the 3rd column shows the corresponding ISIC Rev. 3.1 Code and the 4th column the assigment of each industry to a modified version of the Castellacci taxonomy.

## Appendix B. Mathematical Appendix

***Definition:*** A weighted directed graph *G* consists of a pair , where *V* is a finite and non-empty set of elements *i* called nodes and *E* is a finite set of elements  called edges, with . A weighted directed graph is described by two functions  and to each , a weight  is assigned (see e.g. Harary et al., 1965).

***Derivation of regional innovation flow networks:*** Given the basic concept of a weighted directed graph, to derive for each region *r* the weighted adjacency matrix  from the regional innovation flow matrix in Equation (5), in a first step the right hand side of Equation (4) is plugged into Equation (5), which yields:

(B1)

According to its definition, the Leontief-Inverse  can be written as . Applying Eulerian power series to Equation B1, matrix  is split into different layers as follows:

(B2)

Since by definition an adjacency matrix contains only direct linkages between adjacent nodes, we take the first layer matrix from Equation B2 to derive the adjacency matrix for each region *r* (Harary et al., 1965). To make in a further step structural relationships comparable across regions, the first layer matrix  is normalised:

(B3)

In Equation B3, the normalisation proceeds along total direct innovation flows and each element of  is thus divided by the sum of column- or equivalently row-sums of . Each element  reflects the share of direct innovation flows from node *i* to node *j* in total, system-wide direct innovation flows. This normalisation procedure implies that the focus in the analysis is set on 'purely relational characteristics' as highlighted by Montresor and Vittucci Marzetti (2009, p. 135). Matrix  is already quite similar to the regional weighted adjacency matrices, but still contains self-loops. As a (weighted) directed graph by definition does not include self-loops these have to be corrected for: Let  be an  vector containing all elements from the main diagonal of . Then the weighted adjacency matrix  for a single region *r* reads as:

(B4)

For a single region *r*, a generic element  of the adjacency matrix from Equation B4 corresponds to the share of overall direct innovation flows which industry *i* delivers to industry *j*.

## Appendix

**Table C.1.** Industry strength centrality scores for the three territories, 2009-2010. Based on ABS Data (2013a, 2013b, 2014a, 2015). Author's own calculations.

	In-Strength			Out-Strength		
ANZSIC06 Code	NSW	VIC	QLD	NSW	VIC	QLD
01-05	0.0085	0.008	0.0056	0.0062	0.0034	0.0144
06-10	0.0162	0.0024	0.0045	0.0382	0.0125	0.1834
11	0.0139	0.0171	0.0215	0.0187	0.0381	0.0247
12	0.0086	0.0059	0.0001	0.0023	0.0037	0.0024
13	0.0025	0.0043	0.0012	0.0009	0.0028	0.0009
14	0.0003	0.0003	0.0006	0.0084	0.0037	0.0091
15-16	0.0012	0.0014	0.0007	0.0292	0.0125	0.0012
17	0.0109	0.0085	0.0172	0.0028	0.0126	0.0058
18	0.0082	0.0095	0.0079	0.0144	0.0528	0.0193
19	0.0014	0.0035	0.0017	0.0071	0.0219	0.0055
20	0.0004	0.0003	0.0016	0.0119	0.0111	0.0327
21	0.0156	0.0016	0.0165	0.0238	0.0079	0.0513
22	0.0024	0.0022	0.0042	0.0185	0.0121	0.0233
23	0.0091	0.0293	0.0255	0.004	0.0633	0.0097
24	0.0178	0.009	0.0126	0.0491	0.0661	0.0365
25	0.0032	0.0043	0.0086	0.0009	0.0032	0.0017
26	0.0093	0.0062	0.0376	0.0026	0.0171	0.0075
27-29	0.0065	0.0065	0.0036	0.0076	0.0139	0.0164
30	0.0724	0.0697	0.0802	0.0048	0.0085	0.0261
31	0.0286	0.0277	0.0706	0.0022	0.0059	0.0157
32	0.0237	0.0284	0.0433	0.0152	0.0096	0.0105
33-38	0.0339	0.0384	0.0469	0.0515	0.054	0.0283
39-43	0.0363	0.0479	0.0382	0.0013	0.0067	0.0026
44-45	0.0286	0.0417	0.0361	0.0001	0.0007	0.001
46-53	0.0264	0.0482	0.0281	0.0108	0.0156	0.025
54	0.0039	0.0031	0.001	0.0061	0.0154	0.0032
56	0.003	0.0015	0.0004	0.0025	0.0001	0.0002
57,59	0.0008	0.0004	0.0002	0.0026	0.0018	0.0042
55,58,60	0.0122	0.0187	0.0139	0.0187	0.03	0.006
62	0.0089	0.0094	0.0063	0.3445	0.0853	0.0361
63-64	0.0332	0.0123	0.0108	0.0238	0.0215	0.005
66-67	0.2686	0.1236	0.1428	0.0035	0.0097	0.0025
69	0.0084	0.0073	0.0077	0.0938	0.135	0.156
70	0.0044	0.008	0.0037	0.0622	0.0635	0.1101
72-73,75-77	0.0587	0.0673	0.0684	0.0102	0.0145	0.0051
80-82	0.024	0.0338	0.0348	0.0001	0	0
84-87	0.0606	0.0758	0.0506	0.0001	0.0002	0.0001
89-92	0.0116	0.0257	0.0137	0.0001	0.0022	0.0011
94-96	0.0183	0.035	0.019	0.0021	0.005	0.0034

**Table C.2.** Gini-Indexes for the three territories, 2009-2010. Based on ABS Data (2013a, 2013b, 2014a, 2015). Author's own calculations.

	Gini-Index (In-Strength)			Gini-Index (Out-Strength)		
ANZSIC06 Code	NSW	VIC	QLD	NSW	VIC	QLD
01-05	0.81	0.7701	0.725	0.8675	0.88	0.8845
06-10	0.7834	0.7112	0.7133	0.8381	0.8472	0.7001
11	0.7594	0.6688	0.8335	0.8281	0.8365	0.8568
12	0.7734	0.6656	0.6979	0.9411	0.9432	0.9432
13	0.7243	0.7231	0.7104	0.6785	0.6814	0.6935
14	0.6757	0.6603	0.7211	0.8556	0.8543	0.865
15-16	0.6961	0.737	0.7454	0.635	0.6376	0.6746
17	0.9608	0.9223	0.9725	0.6981	0.6925	0.7336
18	0.7288	0.6732	0.9023	0.6177	0.6212	0.653
19	0.8128	0.8902	0.8302	0.6607	0.6652	0.7388
20	0.7735	0.6964	0.9384	0.8441	0.8463	0.8694
21	0.9423	0.8652	0.9705	0.7738	0.7782	0.7736
22	0.8745	0.7374	0.9139	0.7529	0.757	0.7941
23	0.7905	0.7534	0.8153	0.8266	0.8228	0.83
24	0.8253	0.7398	0.8644	0.6738	0.6917	0.7063
25	0.7945	0.6872	0.8483	0.7092	0.746	0.7523
26	0.8727	0.7566	0.9497	0.6156	0.6361	0.6772
27-29	0.8099	0.7281	0.7199	0.6899	0.694	0.7532
30	0.6979	0.6252	0.7283	0.8218	0.8199	0.8632
31	0.7168	0.6675	0.7716	0.7562	0.756	0.7863
32	0.7092	0.6312	0.7353	0.8579	0.8581	0.8783
33-38	0.7361	0.6452	0.8567	0.5728	0.5844	0.6236
39-43	0.7331	0.6913	0.719	0.6115	0.6103	0.6609
44-45	0.7129	0.7157	0.7561	0.6158	0.614	0.6626
46-53	0.7054	0.7827	0.7213	0.5975	0.6143	0.6335
54	0.864	0.7531	0.7334	0.7236	0.7266	0.7563
56	0.8771	0.8629	0.778	0.8367	0.8383	0.8487
57,59	0.8765	0.871	0.8434	0.6337	0.6488	0.706
55,58,60	0.7967	0.7346	0.8095	0.6326	0.6369	0.6825
62	0.9179	0.8947	0.9337	0.8237	0.8184	0.8717
63-64	0.9576	0.9157	0.9154	0.7287	0.7358	0.7569
66-67	0.9137	0.7998	0.7817	0.674	0.6792	0.6959
69	0.8057	0.7003	0.7773	0.6861	0.6809	0.754
70	0.8216	0.8077	0.8385	0.7672	0.7721	0.7831
72-73,75-77	0.7942	0.7244	0.8125	0.669	0.6583	0.7381
80-82	0.7549	0.6703	0.7464	0.6502	0.6557	0.7052
84-87	0.7594	0.7232	0.7109	0.8044	0.7991	0.8239
89-92	0.709	0.6934	0.6982	0.7274	0.7395	0.7825
94-96	0.7414	0.8143	0.7149	0.6851	0.6743	0.7207

## References

[CIT0001] Andersen E. (2010[1992]) Approaching National Systems of Innovation from the Production and Linkage Structure. In: B.-A. Lundvall (ed.) *National Innovation Systems: Toward a Theory of Innovation and Interactive Learning*. London, Anthem Press, 71–96.

[CIT0002] Arrow K.J. (1962) The Economic Implications of Learning by Doing. *The Review of Economic Studies*, 29, 155–173. doi: 10.2307/2295952

[CIT0003] Asheim B.T. and Coenen L. (2005) Knowledge Bases and Regional Innovation Systems: Comparing Nordic Clusters. *Research Policy*, 34, 1173–1190. doi: 10.1016/j.respol.2005.03.013

[CIT0004] Asheim B. and Gertler M. (2005) The Geography of Innovation: Regional Innovation Systems. In: J. Fagerberg, D. Mowery and R. Nelson (eds.) *The Oxford Handbook of Innovation*. Oxford, Oxford University Press, 291–317.

[CIT0005] Asheim B.T. and Isaksen A. (1997) Location, Agglomeration and Innovation: Towards Regional Innovation Systems in Norway? *European Planning Studies*, 5, 299–330. doi: 10.1080/09654319708720402

[CIT0006] Australian Bureau of Statistics (2008) ANZSIC06, Correspondence Tables 2006. Cat. No. 1292.0.55.005.

[CIT0007] Australian Bureau of Statistics (2010) Research and Experimental Development: All Sector Summary, Australia, 2008–09. Cat. No. 8112.0.

[CIT0008] Australian Bureau of Statistics (2013a) Research and Experimental Development: Businesses, Australia, 2011–12. Cat. No. 8104.0.

[CIT0009] Australian Bureau of Statistics (2013b) Employed Persons by Industry Group (ANZSIC06), Sex, State, Status in Employment, Customised Report.

[CIT0010] Australian Bureau of Statistics (2014a) Australian National Accounts: State Accounts, 2012–13. Cat. No. 5220.0.

[CIT0011] Australian Bureau of Statistics (2014b) Australian System of National Accounts, Concepts, Sources and Methods. Cat. No. 5216.0.

[CIT0012] Australian Bureau of Statistics (2015) Australian National Accounts: Input-Output Tables, 2009–10. Cat. No. 5209.0.55.001.

[CIT0013] Australian Government Department of Industry and Science (2010) Australian Innovation System Report 2010. http://www.industry.gov.au/Office-of-the-Chief-Economist/Publications/Pages/Australian-Innovation-System.aspx#, accessed 2015/08/06.

[CIT0014] Australian Government, Department of Innovation, Industry, Science and Research (2011) Focusing Australia's Publicly Funded Research Review, The States and Territories in Australia's Research System: Their Roles and Intersection with the Commonwealth: Appendix B. http://www.industry.gov.au/research/Documents/AppendixB-StatesAndTerritories-ResearchStrategiesAndPriorities.pdf, accessed 2015/08/06.

[CIT0015] Autio E. (1998) Evaluation of RTD in Regional Systems of Innovation. *European Planning Studies*, 6, 131–140. doi: 10.1080/09654319808720451

[CIT0016] Becattini G. 2004[1989] *Industrial Districts: A New Approach to Industrial Change*. Cheltenham, Edward Elgar Publishing.

[CIT0017] Borgatti S.P. (2005) Centrality and Network Flow. *Social Networks*, 27, 55–71. doi: 10.1016/j.socnet.2004.11.008

[CIT0018] Brachert M., Brautzsch H.-U. and Titze M. (2016) Mapping Potentials for Input-Output-Based Innovation Flows in Industrial Clusters – An Application to Germany. *Economic Systems Research*, 28, 450–466. doi: 10.1080/09535314.2016.1244517

[CIT0019] Castellacci F. (2008) Technological Paradigms, Regimes and Trajectories: Manufacturing and Service Industries in a New Taxonomy of Sectoral Patterns of Innovation. *Research Policy*, 37, 978–994. doi: 10.1016/j.respol.2008.03.011

[CIT0020] Cooke P. (1992) Regional Innovation Systems: Competitive Regulation in the New Europe. *Geoforum*, 23, 365–382. doi: 10.1016/0016-7185(92)90048-9

[CIT0021] Cooke P. (2001) Regional Innovation Systems, Clusters, and the Knowledge Economy. *Industrial and Corporate Change*, 10, 945–974. doi: 10.1093/icc/10.4.945

[CIT0022] Cooke P. (2005) Regionally Asymmetric Knowledge Capabilities and Open Innovation: Exploring ‘Globalisation 2’ – A New Model of Industry Organisation. *Research Policy*, 34, 1128–1149. doi: 10.1016/j.respol.2004.12.005

[CIT0023] Cooke P., Gomez Uranga M. and Etxebarria G. (1997) Regional Innovation Systems: Institutional and Organisational Dimensions. *Research Policy*, 26, 475–491. doi: 10.1016/S0048-7333(97)00025-5

[CIT0024] De la Mothe J. and Paquet G. (eds.) (1998) *Local and Regional Systems of Innovation*. New York, Kluwer Academic Publishers.

[CIT0025] DeBresson C. (1996) Why Innovative Activities Cluster. In: C. DeBresson (ed.) *Economic Interdependence and Innovative Activity*. Cheltenham, Edward Elgar Publishing, 149–164.

[CIT0026] Doloreux D. and Parto S. (2004) Regional Innovation Systems: A Critical Synthesis. The United Nations University, UNU-INTECH Discussion Papers. http://www.intech.unu.edu/publications/discussion-papers/2004-17.pdf, accessed 2015/08/06.

[CIT0027] Edquist C. (2012[1997]) Introduction. In: C. Edquist (ed.) *Systems of Innovation: Technologies, Institutions and Organizations*. London, Routledge, 1–35.

[CIT0028] Fagerberg J., Mowery D.C. and Verspagen B. (2008) Innovation-systems, Path-dependency and Policy: The Co-evolution of Science, Technology and Innovation Policy and Industrial Structure in a Small, Resource-based Economy. DIME Working paper 2008, http://www.dime-eu.org/files/active/0/FagMowVer.pdf, accessed at 2017/01/30.

[CIT0029] Fagiolo G. (2007) Clustering in Complex Directed Networks. *Physical Review E*, 76, 026107. doi: 10.1103/PhysRevE.76.026107 17930104

[CIT0030] Florida R. (1995) Toward the Learning Region. *Futures*, 27, 527–536. doi: 10.1016/0016-3287(95)00021-N

[CIT0031] Freeman L.C. (1978–1979) Centrality in Social Networks Conceptual Clarification. *Social Networks*, 1, 215–239. doi: 10.1016/0378-8733(78)90021-7

[CIT0032] Gregory R. (1993) The Australian Innovation System. In: R. Nelson (ed.) *National Innovation Systems: A Comparative Analysis*. Oxford, Oxford University Press, 324–352.

[CIT0033] Harary F., Norman R. and Cartwright D. (1965) *Structural Models: An Introduction to the Theory of Directed Graphs*. New York, John Wiley & Sons, Inc.

[CIT0034] Hassan S., Bucifal S., Drake P. and Hendrickson L. (2015) Australian Geography of Innovative Entrepreneurship. Research Paper 3/2015. Department of Industry, Innovation and Science, Office of the Chief Economist, Canberra.

[CIT0035] Hauknes J. and Knell M. (2009) Embodied Knowledge and Sectoral Linkages: An Input-Output Approach to the Interaction of High- and Low-Tech Industries. *Research Policy*, 38, 459–469. doi: 10.1016/j.respol.2008.10.012

[CIT0036] Hirschman A. (1958) *The Strategy of Economic Development*. New Haven, CT, Yale University Press.

[CIT0037] Howells J. (1999) Regional Systems of Innovation? In: D. Archibugi, J. Howells and J. Michie (eds.) *Innovation Policy in a Global Economy*. Cambridge, Cambridge University Press, 67–93.

[CIT0038] Jacobs J. (1969) *The Economy of Cities*. New York, Random House.

[CIT0039] Junius T. and Oosterhaven J. (2003) The Solution of Updating or Regionalizing a Matrix with Both Positive and Negative Entries. *Economic Systems Research*, 15, 87–96. doi: 10.1080/0953531032000056954

[CIT0040] Kalmbach P. and Kurz H. (1985) Internationale Wettbewerbsfähigkeit und Technologieintensität: Eine Anwendung des Subsystem-Ansatzes. *IFO-Studien Zeitschrift für empirische Wirtschaftsforschung*, 31, 149–168.

[CIT0041] Leoncini R. and Montresor S. (2000) Network Analysis of Eight Technological Systems. *International Review of Applied Economics*, 14, 213–234. doi: 10.1080/02692170050024750

[CIT0042] Leoncini R. and Montresor S. (2003) *Technological Systems and Intersectoral Innovation Flows*. Cheltenham, Edgward Elgar Publishing.

[CIT0043] Leoncini R., Maggioni M.A. and Montresor S. (1996) Intersectoral Innovation Flows and National Technological Systems: Network Analysis for Comparing Italy and Germany. *Research Policy*, 25, 415–430. doi: 10.1016/0048-7333(95)00843-8

[CIT0044] Maillat D. (1995) Territorial Dynamic, Innovative Milieus and Regional Policy. *Entrepreneurship & Regional Development: An International Journal*, 7, 157–165. doi: 10.1080/08985629500000010

[CIT0045] Marengo L. and Sterlacchini A. (1990) Intersectoral Technology Flows: Methodological Aspects and Empirical Applications. *Metroeconomica*, 41, 19–39. doi: 10.1111/j.1467-999X.1990.tb00455.x

[CIT0046] Miller R. and Blair P. (2009) *Input-Output Analysis: Foundations and Extensions*, 2nd ed Cambridge, Cambridge University Press.

[CIT0047] Momigliano F. and Siniscalco D. (1982) Note in Tema di Terziarizzazione e Deindustrializzazione. *Moneta e Credito*, 35, 143–181.

[CIT0048] Montresor S. and Vittucci Marzetti G. (2008) Innovation Clusters in Technological Systems: A Network Analysis of 15 OECD Countries for the Middle '90s. *Industry and Innovation*, 15, 321–346. doi: 10.1080/13662710802041679

[CIT0049] Montresor S. and Vittucci Marzetti G. (2009) Applying Social Network Analysis to Input-Output Based Innovation Matrices: An Illustrative Application to Six OECD Technological Systems for the Middle 1990s. *Economic Systems Research*, 21, 129–149. doi: 10.1080/09535310902940228

[CIT0050] Morgan K. (1997) The Learning Region: Institutions, Innovation and Regional Renewal. *Regional Studies*, 31, 491–503. doi: 10.1080/00343409750132289

[CIT0051] Nelson R. (ed.) (1993) *National Innovation Systems: A Comparative Analysis*. Oxford, Oxford University Press.

[CIT0052] Newman M.E.J. (2004) Analysis of Weighted Networks. *Physical Review E*, 70, 056131. 10.1103/PhysRevE.70.056131 15600716

[CIT0053] Onnela J.-P., Saramäki J., Kertész J. and Kaski K. (2005) Intensity and Coherence of Motifs in Weighted Complex Networks. *Physical Review E*, 71, 065103. doi: 10.1103/PhysRevE.71.065103 16089800

[CIT0054] Papaconstantinou G., Sakurai N. and Wyckoff A. (1998) Domestic and International Product-Embodied R&D Diffusion. *Research Policy*, 27, 301–314. doi: 10.1016/S0048-7333(98)00044-4

[CIT0055] Pavitt K. (1984) Sectoral Patterns of Technical Change: Towards a Taxonomy and a Theory. *Research Policy*, 13, 343–373. doi: 10.1016/0048-7333(84)90018-0

[CIT0056] Porter M. and Stern S. (2002) National Innovative Capacity. *The Global Competitiveness Report*, (2001/02)/102-118.

[CIT0057] Romer P. M. (1986) Increasing Returns and Long-Run Growth. *Journal of Political Economy*, 94, 1002–1037. doi: 10.1086/261420

[CIT0058] Stone R. (1961) *Input-Output and National Accounts*. Paris, Organization for Economic Cooperation and Development.

[CIT0059] Strohmaier R. (2014) Innovations and Economic Change – The Role of General Purpose Technologies, a Methodological and Empirical Analysis. PhD thesis, University of Graz, Austria.

[CIT0060] Uyarra E. (2010) What Is Evolutionary about ‘Regional Systems of Innovation’? Implications for Regional Policy. *Journal of Evolutionary Economics*, 20, 115–137. doi: 10.1007/s00191-009-0135-y

