# Supplementary material for: Australia's regional innovation systems: inter-industry interaction in innovative activities in three Australian territories
Source: Econ Syst Res. 2017 Apr 3;29(3):357–84. doi: 10.1080/09535314.2017.1301886 (PMC5633018; doi:10.1080/09535314.2017.1301886)
Supplement: Supplement_Material.pdf [file cesr_a_1301886_sm6015.pdf]

Industry	ANZSIC06 Code	ISIC Rev. 3.1 Code	Castellacci Taxonomy (modified)
Agriculture, Forestry and Fishing	01-05	01-05	ERB
Mining	06-10	10-14	ERB
Food Product Manufacturing	11	15	SDG
Beverage and Tobacco Product Manufacturing	12	15, 16	SDG
Textile, Leather, Clothing and Footwear Manufacturing	13	17-19	SDG
Wood Product Manufacturing	14	20	SDG
Pulp, Paper and Converted Paper Product Manufacturing Printing (including the Reproduction of Recorded Media)	15-16	21-22	SDG
Petroleum and Coal Product Manufacturing	17	23	ERB
Basic Chemical and Chemical Product Manufacturing	18	24	SCB
Polymer Product and Rubber Product Manufacturing	19	25	SCI
Non-Metallic Mineral Product Manufacturing	20	26	SCI
Primary Metal and Metal Product Manufacturing	21	27	SCI
Fabricated Metal Product Manufacturing	22	28	SCI
Transport Equipment Manufacturing	23	34-35	SCI
Machinery and Equipment Manufacturing	24	29-32	SCB
Furniture and Other Manufacturing	25	36	SDG
Electricity Supply	26	40	OI
Gas Supply; Water Supply, Sewerage and Drainage Services; Waste Collection, Treatment and Disposal Services	27-29	40, 41, 90	OI
Building Construction	30	45	OI
Heavy and Civil Engineering Construction	31	45	OI
Construction Services	32	70,45	OI
Wholesale Trade	33-38	50-51	PIS
Retail Trade	39-43	50,52	SDS
Accommodation and Food Services	44-45	55	SDS
Transport, Postal and Warehousing	46-53	60-64	PIS
Publishing (except Internet and Music Publishing)	54	22,74,72	SDS
Broadcasting (except Internet)	56	92	SDS
Internet Publishing and Broadcasting; Internet Service Providers, Web Search Portals and Data Processing Services	57, 59	72	SDS
Motion Picture and Sound Recording Activities; Telecommunication Services; Library and Other Information Services	55, 58, 60	64, 72, 74, 92	NIS
Finance	62	65	NIS
Insurance and Superannuation Funds, Auxiliary Finance and Insurance Services	63-64	66-67	NIS
Rental, Hiring and Real Estate Services	66-67	70-71	NIS
Professional, Scientific and Technical Services (Except Computer System Design and Related Services)	69	73-74	KIBS
Computer System Design and Related Services	70	72	KIBS
Administration Services and Support Services; Public Administration and Safety	72-73, 75-77	74-75	OI
Education and Training	80-82	80	OI
Health Care and Social Assistance	84-87	85	OI
Arts and Recreation Services	89-92	92	SDS
Other Services	94-96	93-97	OI

Table A1: Merged Industry Classification based on ANZSIC06.
Note that the 3rd column shows the corresponding ISIC Rev. 3.1 Code
and the 4th column the assignment of each industry to a modified version of the Castellacci taxonomy.

	In-Strength			Out-Strength		
ANZSIC06 Code	NSW	VIC	QLD	NSW	VIC	QLD
01-05	0.0085	0.008	0.0056	0.0062	0.0034	0.0144
06-10	0.0162	0.0024	0.0045	0.0382	0.0125	0.1834
11	0.0139	0.0171	0.0215	0.0187	0.0381	0.0247
12	0.0086	0.0059	0.0001	0.0023	0.0037	0.0024
13	0.0025	0.0043	0.0012	0.0009	0.0028	0.0009
14	0.0003	0.0003	0.0006	0.0084	0.0037	0.0091
15-16	0.0012	0.0014	0.0007	0.0292	0.0125	0.0012
17	0.0109	0.0085	0.0172	0.0028	0.0126	0.0058
18	0.0082	0.0095	0.0079	0.0144	0.0528	0.0193
19	0.0014	0.0035	0.0017	0.0071	0.0219	0.0055
20	0.0004	0.0003	0.0016	0.0119	0.0111	0.0327
21	0.0156	0.0016	0.0165	0.0238	0.0079	0.0513
22	0.0024	0.0022	0.0042	0.0185	0.0121	0.0233
23	0.0091	0.0293	0.0255	0.004	0.0633	0.0097
24	0.0178	0.009	0.0126	0.0491	0.0661	0.0365
25	0.0032	0.0043	0.0086	0.0009	0.0032	0.0017
26	0.0093	0.0062	0.0376	0.0026	0.0171	0.0075
27-29	0.0065	0.0065	0.0036	0.0076	0.0139	0.0164
30	0.0724	0.0697	0.0802	0.0048	0.0085	0.0261
31	0.0286	0.0277	0.0706	0.0022	0.0059	0.0157
32	0.0237	0.0284	0.0433	0.0152	0.0096	0.0105
33-38	0.0339	0.0384	0.0469	0.0515	0.054	0.0283
39-43	0.0363	0.0479	0.0382	0.0013	0.0067	0.0026
44-45	0.0286	0.0417	0.0361	0.0001	0.0007	0.001
46-53	0.0264	0.0482	0.0281	0.0108	0.0156	0.025
54	0.0039	0.0031	0.001	0.0061	0.0154	0.0032
56	0.003	0.0015	0.0004	0.0025	0.0001	0.0002
57,59	0.0008	0.0004	0.0002	0.0026	0.0018	0.0042
55,58,60	0.0122	0.0187	0.0139	0.0187	0.03	0.006
62	0.0089	0.0094	0.0063	0.3445	0.0853	0.0361
63-64	0.0332	0.0123	0.0108	0.0238	0.0215	0.005
66-67	0.2686	0.1236	0.1428	0.0035	0.0097	0.0025
69	0.0084	0.0073	0.0077	0.0938	0.135	0.156
70	0.0044	0.008	0.0037	0.0622	0.0635	0.1101
72-73,75-77	0.0587	0.0673	0.0684	0.0102	0.0145	0.0051
80-82	0.024	0.0338	0.0348	0.0001	0	0
84-87	0.0606	0.0758	0.0506	0.0001	0.0002	0.0001
89-92	0.0116	0.0257	0.0137	0.0001	0.0022	0.0011
94-96	0.0183	0.035	0.019	0.0021	0.005	0.0034

Table C1: Industry strength centrality scores for the three territories, 2009-2010. Based on ABS Data (2013a, 2013b, 2014a, 2015). Author's own calculations.

	Gini-Index (In-Strength)			Gini-Index (Out-Strength)		
ANZSIC06 Code	NSW	VIC	QLD	NSW	VIC	QLD
01-05	0.81	0.7701	0.725	0.8675	0.88	0.8845
06-10	0.7834	0.7112	0.7133	0.8381	0.8472	0.7001
11	0.7594	0.6688	0.8335	0.8281	0.8365	0.8568
12	0.7734	0.6656	0.6979	0.9411	0.9432	0.9432
13	0.7243	0.7231	0.7104	0.6785	0.6814	0.6935
14	0.6757	0.6603	0.7211	0.8556	0.8543	0.865
15-16	0.6961	0.737	0.7454	0.635	0.6376	0.6746
17	0.9608	0.9223	0.9725	0.6981	0.6925	0.7336
18	0.7288	0.6732	0.9023	0.6177	0.6212	0.653
19	0.8128	0.8902	0.8302	0.6607	0.6652	0.7388
20	0.7735	0.6964	0.9384	0.8441	0.8463	0.8694
21	0.9423	0.8652	0.9705	0.7738	0.7782	0.7736
22	0.8745	0.7374	0.9139	0.7529	0.757	0.7941
23	0.7905	0.7534	0.8153	0.8266	0.8228	0.83
24	0.8253	0.7398	0.8644	0.6738	0.6917	0.7063
25	0.7945	0.6872	0.8483	0.7092	0.746	0.7523
26	0.8727	0.7566	0.9497	0.6156	0.6361	0.6772
27-29	0.8099	0.7281	0.7199	0.6899	0.694	0.7532
30	0.6979	0.6252	0.7283	0.8218	0.8199	0.8632
31	0.7168	0.6675	0.7716	0.7562	0.756	0.7863
32	0.7092	0.6312	0.7353	0.8579	0.8581	0.8783
33-38	0.7361	0.6452	0.8567	0.5728	0.5844	0.6236
39-43	0.7331	0.6913	0.719	0.6115	0.6103	0.6609
44-45	0.7129	0.7157	0.7561	0.6158	0.614	0.6626
46-53	0.7054	0.7827	0.7213	0.5975	0.6143	0.6335
54	0.864	0.7531	0.7334	0.7236	0.7266	0.7563
56	0.8771	0.8629	0.778	0.8367	0.8383	0.8487
57,59	0.8765	0.871	0.8434	0.6337	0.6488	0.706
55,58,60	0.7967	0.7346	0.8095	0.6326	0.6369	0.6825
62	0.9179	0.8947	0.9337	0.8237	0.8184	0.8717
63-64	0.9576	0.9157	0.9154	0.7287	0.7358	0.7569
66-67	0.9137	0.7998	0.7817	0.674	0.6792	0.6959
69	0.8057	0.7003	0.7773	0.6861	0.6809	0.754
70	0.8216	0.8077	0.8385	0.7672	0.7721	0.7831
72-73,75-77	0.7942	0.7244	0.8125	0.669	0.6583	0.7381
80-82	0.7549	0.6703	0.7464	0.6502	0.6557	0.7052
84-87	0.7594	0.7232	0.7109	0.8044	0.7991	0.8239
89-92	0.709	0.6934	0.6982	0.7274	0.7395	0.7825
94-96	0.7414	0.8143	0.7149	0.6851	0.6743	0.7207

Table C2: Gini-Indexes for the three territories, 2009-2010.

Based on ABS Data (2013a, 2013b, 2014a, 2015). Author's own calculations.
